# Differential Influences of Parenting Dimensions and Parental Physical Abuse during Childhood on Overweight and Obesity in Adolescents

**DOI:** 10.3390/children4030017

**Published:** 2017-03-07

**Authors:** Thomas Mößle, Sören Kliem, Anna Lohmann, Marie Christine Bergmann, Dirk Baier

**Affiliations:** 1 Criminological Research Institute of Lower Saxony (Kriminologisches Forschungsinstitut Niedersachsen e.V.), Lützerodestraße 9, 30161 Hannover, Germany; soeren.kliem@kfn.de (S.K.); anna.lohmann@kfn.de (A.L.); Marie.Bergmann@kfn.de (M.C.B.); 2 Institute of Crime and Delinquency, Zurich University of Applied Sciences, Pfingstweidstrasse 96, 8037 Zurich, Switzerland; baid@zhaw.ch

**Keywords:** BMI, body weight, overweight, obesity, parenting style, warmth, monitoring, parental physical abuse, childhood, adolescence

## Abstract

Besides other explanatory variables, parenting styles and parental violence might also be responsible for setting a path towards overweight/obesity in childhood. While this association has consistently been observed for adults, findings for adolescents still remain scarce and inconsistent. Therefore, the goal of this study is to add evidence on this topic for children and adolescents. Analyses are based on a sample of 1729 German, ninth-grade students. To analyze associations between parenting dimensions and weight status, non-parametric conditional inference trees were applied. Three gender-specific pathways for a heightened risk of overweight/obesity were observed: (1) female adolescents who report having experienced severe parental physical abuse and medium/high parental warmth in childhood; (2) male adolescents who report having experienced low or medium parental monitoring in childhood; and (3) this second pathway for male adolescents is more pronounced if the families receive welfare. The importance of promoting parenting styles characterized by warmth and a lack of physical abuse is also discussed. This is one of only a few studies examining the association of parenting dimensions/parental physical abuse and weight status in adolescence. Future studies should include even more parenting dimensions, as well as parental physical abuse levels, in order to detect and untangle gender-specific effects on weight status.

## 1. Introduction

The German Health Interview and Examination Survey for Children and Adolescents (Kinder- und Jugendgesundheitssurvey, KiGGS) reported that 17.1% of German adolescents (aged 14–17) can be classified as overweight or obese. For obesity alone, a prevalence of 8.5% is reported [1]. Besides other explanatory variables, such as genetic factors, nutrition, physical inactivity, sedentary behavior, and impaired sleep patterns [2], parental behavior is also thought to be responsible for setting a path towards overweight and obese children [3]. However, only recently, the focus has shifted from food-related parenting behavior (e.g., portion control, healthy food choice, monitoring of dietary behavior, encouraging/discouraging eating and exercise, etc.) to more general parenting dimensions that focus on the quality of the parent-child relationship [4].

Whereas obesity interventions in childhood have successfully targeted parents [5], as children grow older, parenting behaviors are less strongly associated with outcomes. The likelihood of childhood obesity continuing into adulthood increases in adolescence, hence knowledge regarding this specific age group is eminent. Identifying obesity pathways related to parenting behavior might provide new approaches in health prevention on the parental level. However, findings from pre-adolescent childhood might not always be applicable to adolescents. Indeed, findings on parenting dimensions and obesity in adolescence are often heterogeneous [6]. As the parent-child relationship becomes more complex in adolescence, and with it the associations between parenting behavior and child health outcomes, it is possible that these heterogeneous findings are due to different pathways in different subgroups, as well as smaller effect sizes. Hence, large sample sizes are needed to disentangle these effects.

In the explanation of the link between parenting behavior and obesity, many health behaviors associated with certain parenting dimensions are discussed as protective as well as risk factors. Low parental monitoring, for example, has emerged as an important parenting behavior that is connected to multiple adverse health behaviors in adolescence (e.g., substance abuse, delinquency, and risky sexual behavior). Families characterized by warmth and control, for example, tend to spend more meals together [7], as well as less time in front of screen media [8]. Parental monitoring reduces the amount of less nutrient dense food and increases the number of regular meals [9]. In terms of psychological variables, self-regulation and stress are often regarded as key variables linking parenting to obesity. Low parental warmth might function as a stressor itself and/or leave the child more exposed to other life stressors [6]. Low parental monitoring, as well as low parental responsiveness, might also negatively influence the development of self-regulatory competencies [4,6] regarding nutrition and adaptive coping behavior.

Another important parenting behavior which should not be considered independently of parenting style, as it is particularly relevant for the prediction of various undesirable developments in childhood and adolescence, is parental physical abuse [10,11]. Among the various adverse outcomes of childhood physical abuse, obesity has consistently been linked to childhood exposure to parental violence. In explaining the link between physical maltreatment in childhood and obesity, maladaptive coping responses, stress [12], post-traumatic stress disorder (PTSD) [13], and depression [14] are currently discussed as possible mediators. In a large meta-analysis that analyzed 41 studies on the effect of childhood maltreatment on adult obesity, Danese and Tan [15] found that individuals who suffered from physical abuse in childhood had a 36% higher probability of being obese as adults compared to individuals who did not experience physical abuse in childhood (OR 1.36, CI 95% [1.26,1.47]). The effect was significant even after controlling for childhood socioeconomic status (SES). Additionally, the authors noted that women might be more vulnerable to the effects of maltreatment on obesity [15]. Furthermore, Hemmingsson et al. also confirmed in another meta-analysis a positive dose-response association of violence experienced in childhood and obesity in adults [16].

While this relationship has consistently been observed in studies including adults, findings assessing the relationship of parental physical abuse and adolescent obesity are scarce and inconsistent. In a large three-wave longitudinal study, Shin and Miller found childhood maltreatment effects to be clearly distinguishable only in adults, whereas in adolescents, change in body mass index (BMI) was equal among children with and without a history of physical maltreatment [17]. In their meta-analysis, Danese and Tan report the association between maltreatment and obesity in studies of children and adolescents to be non-significant [15]. In a recent large cohort study (*n* = 10,464), Stensland et al. found a robust and significant relationship between interpersonal violence and increased BMI for both genders in adolescence [18]. After controlling for pubertal development, SES, and lifestyle, this effect was, however, more evident in girls [18].

The aim of the present study is to continue to explore various parenting dimensions and their link to obesity. We employed a data-driven model based on a large representative sample that incorporates gender, parental warmth, parental monitoring, parental physical abuse, SES (welfare dependency), as well as immigrant backgrounds. With the help of recursive binary partitioning, subgroups with the highest likelihood of producing significant differences regarding weight status are uncovered.

## 2. Material and Methods

### 2.1. Participants and Procedure

**Figure 1:**
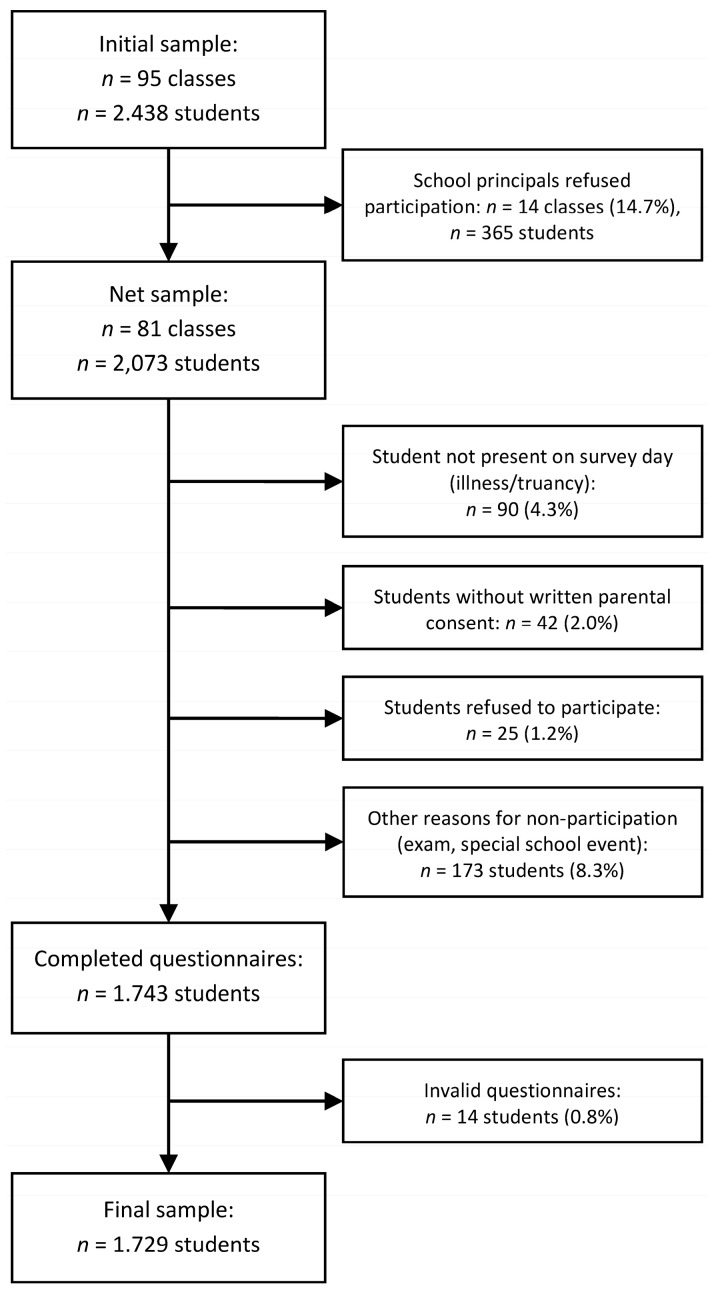
Flowchart of sampling procedure and reasons for non-participation.

The sample was obtained from a ninth-grade school survey in Aachen, Germany. Aachen is located in the tristate-area of Germany, Belgium and the Netherlands, has a population of about 240,000 and a ratio of immigrants of one out of three (33%). The school survey was audited and approved by the state school authorities. With this method of collection, no further ethical approval was required. American Psychological Association (APA) ethical standards were followed in the conduct of the study. From the overall population of 95 Aachen ninth-grade classes (2438 students), 14 classes refused participation (14.7%), resulting in a sample of 81 classes, comprised of 2073 students. On survey day, 330 students did not participate due to various reasons (Figure 1). Some questionnaires (*n* = 14) were excluded because they were clearly invalid (e.g., first response always checked), resulting in 1729 valid datasets. Considering non-participation on the school level, as well as non-participation by individual student participation, the overall response rate was 70.9% (without considering non-participating schools: 83.4%). The average age of participants was 14.9 years (*SD* = 0.73). About half of the study participants were male (51.6%) and 43.3% of the students were from immigrant backgrounds.

Data collection was carried out in March 2014 within the classroom setting. The study was designed as a criminological self-report study targeting low base-rate phenomena of deviant behavior in youths and its possible predictors. In addition to a variety of social topics, such as family, friends, leisure time, media usage, school experiences, substance use, juvenile delinquency, and victimization, physical factors such as body height and weight were also assessed. A prerequisite for participation was written parental consent, albeit students could also independently refuse participation. All questionnaire data was obtained by trained interviewers. Lastly, all questionnaires used were previously validated in large German surveys of adolescents.

### 2.2. Measures

#### 2.2.1. Body Mass Index (BMI)

Students´ body height (cm) and weight (kg) was assessed via self-reporting. BMI was calculated as weight in kg/(height in m)^2^. BMI scores were categorized according to age and gender-specific norms for children and adolescents aged 0–18 by Kromeyer-Hausschild and colleagues [19]. Students with BMI percentiles >90–97 were categorized as overweight, and students with BMI percentiles >97 were categorized as obese. Descriptive sample statistics are provided in Table 1.

#### 2.2.2. Parental Physical Abuse

To assess parental physical abuse during childhood, a German retrospective short version of the Conflict Tactic Scale 1 was used [11,20]. Students were asked whether they had been physically abused by their mothers or fathers during childhood (prior to the age of 12; six-point scale: *never, once or twice*, *3 to 12 times*, *several times a month*, *once a week*, *several times a week*). Students reporting being *slapped or spanked*, *pushed, grabbed or shoved,* or *thrown something at*, at least once by either father or mother, were categorized as having experienced moderate parental physical abuse. Students reporting being *hit with something*, *hit with a fist*, or *beaten up* at least once by either father or mother, were categorized as having experienced severe parental physical abuse (*no* = 0, *moderate* = 1, *severe* = 2). This coding is in accordance with a procedure by Loh et al. [21] and has also been validated in a large representative German study of adolescents [11]. It is based on severity of punitive behavior, rather than frequency.

#### 2.2.3. Parenting Style—Warmth and Monitoring

Parental warmth and monitoring were each assessed on a separate scale. Students were asked to rate their mother’s and father’s behavior during their childhood (prior to the age of 12) on a five-point scale (1 = *never*, 2 = *seldom*, 3 = *sometimes*, 4 = *often*, 5 = *very often*). The six items to measure warmth were: “*praised me, if I did something well*”; “*really looked after me*”; “*comforted me when I was sad*”; “*calmed me when I was scared*”; “*hugged me*”; “*undertook something with me”.* The three items to measure monitoring were: “*precisely knew where I was*”; “*made sure when I was home in the evening*”; “*asked who I was friends with*”. Internal consistencies for each scale were for the fathers Cronbachs α_warmth_ = 0.89, Cronbachs α_monitoring_ = 0.76 and for the mothers Cronbachs α_warmth_ = 0.85, Cronbachs α_monitoring_ = 0.64 (These values are comparable to those of another large German survey of adolescents; see Donath et al. [11]). The mean score for each parent was computed, then, based on the combined mean scores, the following classifications for warmth and monitoring were assigned: 1–3 = low (indicating that *sometimes* was the possible highest response chosen for all items), >3–4.5 = medium (indicating that *often* was chosen at least once), >4.5–5 = high (indicating that *very often* was predominantly chosen).

#### 2.2.4. Socioeconomic Status (SES) and Immigrant Backgrounds

For the assessment of SES, students were asked whether their parents received any form of social welfare payments by the state. Students were classified as having an immigrant background if at least one parent was born in a country other than Germany.

### 2.3. Data Analysis

Regarding missing values, all students with missing values on either body weight (*n* = 197) and body height (*n* = 109), or age (*n* = 6) and gender (*n* = 5), as prerequisites for determining BMI, were excluded (*n* = 211, 12.2%). The proportion of missing values on item level after this exclusion was <1.0%. We then applied chained equation modeling [22], using the following variables: gender, age, BMI >P90 (overweight/obese), as well as parental physical abuse, warmth and monitoring in childhood, welfare dependency, and immigrant background. To avoid non-existing item values, the estimated values (ŷ) were corrected by predictive mean matching (i.e., the observable values closest to the predicted value were chosen). We used the R-package “mice” for this analysis [23].

To analyze associations between parenting behavior (parental monitoring, warmth and physical abuse) and BMI classification (>P90: overweight/obese), non-parametric conditional inference trees (C-Trees) controlling for gender, immigrant background, and SES (welfare dependency) were applied [24,25]. For this analysis, the two BMI groups were merged into one group— overweight/obese. The C-Tree algorithm, which is based on the principle of recursive partitioning, tests the null hypothesis of independence between predictor (gender, parental monitoring, warmth and physical abuse) and response (BMI >P90) variables. Using a permutation test framework, comparable to a stepwise regression, non-significant predictors are excluded [26]. The stop criterion was based on univariate *p* < 0.05. Since permutation tests derive the *p*-values from sample-specific permutation distributions of the test statistics, only *p*-values are reported. The R package “party” (a laboratory for recursive partitioning) was used for this analysis [27].

## 3. Results

### 3.1. Descriptive Statistics

Table 1 provides sample characteristics (*M*, *SD*, *%*) on the predictor and response variables by gender. With 12.0%, male students show a higher proportion of overweight (7.3%) and obesity (4.7%) compared to female students (6.9%, overweight = 5.0%, obesity = 1.9%). There were no gender differences with regard to experiencing parental physical abuse in childhood: 34.7% moderate, 12.3% severe. Female students, however, reported having experienced more parental warmth (high: 43.1%) and monitoring (high: 38.5%) in childhood than male students (warmth high: 34.1%, monitoring high: 29.0%). Odds ratios regarding BMI >P90 are as follows: welfare dependency: OR 1.78 (CI 95% [1.15,2.76]), immigrant background OR 1.70 (CI 95% [1.21,2.41]), male gender OR 1.83 (CI 95% [1.28,2.62]), moderate or severe physical abuse OR 1.4 (CI 95% [1.0,1.98]). Of the 655 students with immigrant background, 147 families also received welfare (22.4%).

### 3.2. Zero-Order Correlations

The zero-order correlations of all predictor and response variables, split by gender, can be found in Table 2. First, positive correlations between the two predictor variables of parenting dimensions were of medium size for male (*r* = 0.48) and female (*r* = 0.45) students. Second, there was a moderate negative correlation of parental warmth and parental physical abuse comparable in extent for male (*r* = −0.32) as well as female (*r* = −0.36) students. Third, parental monitoring was negatively correlated with parental physical abuse to a lesser degree (male: *r* = −0.18; female: *r* = −0.24). Fourth, there were small negative correlations between parenting dimensions and being overweight/obese (warmth: *r* = −0.09; monitoring: *r* = −0.11), but no significant association between parental physical abuse and the response variable, for male students. For female students, no significant correlations between the three predictor variables and being overweight/obese can be observed.

### 3.3. Non-Parametric Conditional Inference Trees

**Figure 2:**
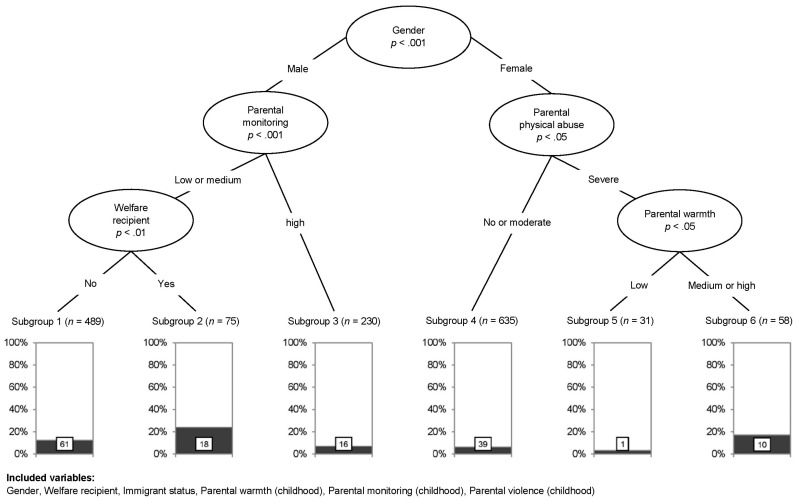
Conditional inference tree plot predicting adolescent overweight/obesity by gender, immigrant background, socioeconomic status (SES; welfare dependency), parental physical abuse, warmth and monitoring in childhood (*n* = 1518). The rectangles on the bottom represent the six subgroups of students computed by C-Tree (the share of overweight/obese students is marked in black). Summarizing the overweight/obese students in the six subgroups results in the total number of overweight/obese students (*n* = 145) in the sample. The three subgroups with a heightened risk of overweight/obesity are subgroups 1, 2 and 6. Subgroup 1 and 2 represent male students having experienced low or medium parental monitoring in childhood. Male adolescents from Subgroup 2 additionally have families who depend on welfare. Subgroup 6 represents female students having experienced severe parental physical abuse and medium or high parental warmth in childhood.

Associations between the parenting dimensions (parental monitoring, warmth, and physical abuse) and the BMI classification (>P90: overweight/obese), controlling for gender, immigrant background, and SES (welfare dependency), were tested using non-parametric conditional inference trees (Figure 2). The first split was introduced for gender, the variable with the strongest association with BMI classification (>P90: overweight/obese); a higher percentage of boys were overweight/obese. There are three pathways of special interest for explaining a heightened risk of overweight/obesity. The right side of Figure 2 shows the female pathway. Female students who report having experienced severe parental physical abuse (*p* < 0.05) and, in addition, medium or high parental warmth in childhood (*p* < 0.05) have a heightened risk of overweight/obesity in adolescence (17.5% vs. 9.6%). The left side of Figure 2 shows two distinct pathways for male students. Male students who report having experienced low or medium parental monitoring in childhood (*p* < 0.001) have a heightened risk of overweight/obesity in adolescence (14% vs. 7%). This is even more pronounced in those from low SES families (24% vs. 12.5%).

## 4. Discussion

In the present study, the association of general parenting behavior in childhood and adolescent weight status was analyzed. General parenting behavior was assessed (a) along the two dimensions of warmth and monitoring, and (b) by parental physical abuse in childhood. Weight status was operationalized by being overweight or obese. With a proportion of 9.6% being classified as overweight (including obese) and a proportion of 3.4% being classified as obese, the results of our sample lie below the prevalence rates of the representative German Health Interview and Examination Survey for Children and Adolescents (Kinder- und Jugendgesundheitssurvey, KiGGS), which reported a prevalence rate of 17.1% in 14- to 17-year-old children and adolescents who are classified as overweight (including obese) and a share of 8.5% classified as obese [1]. One reason for this discrepancy might be our sample’s use of self-reporting to measure weight and height status, compared to the standardized measurement in KIGGS. Self-report measures are, however, not uncommon in non-cohort studies with rather large sample sizes [15,28].

For male students, no influence of parental physical abuse in childhood on overweight or obesity in adolescence was observed, a finding which is also reflected (for male adolescents) in the meta-analytic results by Danese and Tan [15]. The differential influence of physical abuse on male and female students can possibly be explained by a higher tolerance of boys regarding the experience of violence. Girls have been shown to react more strongly to harsh parenting, for example in terms of higher delinquency rates [29] or higher mental health problems [30]. Additionally, depression has been linked to being overweight, which has been explained via the mediation of cortisol activity; however, these findings are only relevant in girls [31]. Cortisol activity in response to stress has also been shown to increase in individuals with a history of maltreatment. The increase in cortisol reactivity has been shown to be higher for individuals suffering from less severe depression [32]. Hence, our findings could reasonably be explained by the following logic: girls who have been physically abused and subjected to parental warmth suffer from less depression, hence their cortisol reactivity to stress is higher, often resulting in a higher BMI. This explanatory pathway is of course highly tentative, but could suggest that underlying physiological mechanisms can and should also be taken into account in further (experimental) studies. Furthermore, parental inconsistency, which could arguably be the case with warm but abusive parents, has exhibited the highest effect size in association with weight (overweight or obesity) in the meta-analysis by Pinquardt [3]. As argued above, these inconsistencies could prevent stress habituation.

However, a lack of general parental monitoring seems to heighten the risk of overweight or obesity for male adolescents [33,34]. Regarding parental monitoring, which seems of higher relevance in males for the prevention of obesity, we would like to offer a rationale using the social identity theory. In males, and especially in adolescents, risk-taking and unhealthy behavior is role-affirmative regarding a masculine in-group [35]. For adolescent boys, especially those from lower socioeconomic background, eating unhealthy and supersized meals is also identity-affirmative in-group behavior, whereas for girls, portion control and healthy eating choices are not only acceptable but are also congruent with acceptable views of femininity.

The subgroup of male adolescents whose families additionally depend on welfare had the highest risk of being overweight or obese. Approximately one-fourth of male youths with low or medium monitoring and low SES were either overweight or obese. Immigrant background, however, did not cause any significant partitioning; hence, it is likely that the association of immigrant background and obesity status can be fully explained by parenting style and SES. The rationale behind the pathway of low parental monitoring and low SES might be that adolescent boys from low SES families have access to unhealthier food choices compared to adolescents from higher SES families.

The interrelationship of parenting dimensions and parental physical abuse in childhood, as observed in this study, might aid to resolve ambiguity in the reported associations of parenting dimensions and weight status, as well as parental physical abuse and weight status [3,15,16,17,33,34,36,37].

### Limitations

Despite having identified differential influences of parental warmth, parental monitoring, and parental physical abuse in childhood on adolescent overweight and obesity, there are certain limitations to the presented results. First, having drawn a large representative sample of ninth-grade students from the city of Aachen, the results are representative only of Aachen children within this age group. However, there is no reason to assume that Aachen adolescents are substantially different from adolescents of other cities with comparable sizes [38]. Nevertheless, the sample is extremely homogenous regarding age, and therefore the results likely reflect associations related to a specific pubescent stage [15]. As the development in male and female adolescents varies, especially in the age spectrum analyzed in this study, gender differences might also reflect developmental differences.

Second, having assessed all variables of interest via self-reporting, some results might differ if other instruments or procedures, such as measuring body weight and height, had been used instead. BMI classifications based on self-reported data, however, have been shown to accurately identify overweight/obesity in young people—despite an underestimation of weight [39]. Third, parenting dimensions and parental physical abuse were assessed only in retrospect. Participants referred to life events up to at least one and on average at least three years prior to the point of assessment, while participants indicated their current height and weight. Although assessed simultaneously, the parenting variables precede the weight-related data. Nevertheless, with these limitations in mind, the present study provided evidence for the association of parenting dimensions/parental physical abuse and overweight and obesity in childhood and adolescence. Large longitudinal studies have also confirmed the expected directionality of the association [40].

Fourth, in a large sample of adolescents, Mellin et al. [41] found that parental monitoring often shows a curvilinear association (inverted U shape) with various health-related behaviors, indicating that a moderate level of monitoring might be ideal. As the model applied in this study only allows binary splits, this kind of association cannot be uncovered. Future studies should employ methods that can model non-linear associations, as many parenting-related as well as food- and dietary-related variables share the notion that moderation might be key.

Fifth, Subgroup 5, containing female adolescents with a history of severe physical abuse and low parental warmth in childhood, is small and consequently possibly spurious. As the method in this study is data-driven, this specific pathway, as well as the others, can only be viewed as a tentative model that should be replicated, including hypothesis testing models.

Whether physical maltreatment, parental monitoring, and parental warmth constitute precursors or accelerating or sustaining factors in weight-related trajectories is also a subject for further investigation. Furthermore, it is uncertain whether the more general parenting concepts investigated within this paper only serve as proxies for more specific nutrition-related parenting. The model presented in this paper can only provide a starting point for the development of more complex hypotheses.

## 5. Conclusions

The association of parental physical abuse and obesity has consistently been observed in studies including adults. Findings assessing this relationship in adolescence are scarce and inconsistent. At least the latter is also true for studies examining the association of parenting dimensions in childhood and adolescent weight status. This study is one of the few looking at parenting dimensions/parental physical abuse in childhood and their association with adolescent weight status. Individual differences in the influence of parental warmth, monitoring, and physical abuse in childhood, as well as their interrelations with adolescent weight status depending on gender, were observed. In future studies, efforts should be made to introduce both parenting dimensions and parental physical abuse to detect and untangle gender-specific effects on overweight and obesity. With regard to health care professionals, the importance of promoting parenting characterized by warmth and a lack of physical abuse is underlined. Changes in parenting behavior can not only strengthen children´s social competencies and lower their risk of delinquency and alcohol or drug abuse, but these changes might also lower the risk of becoming overweight or obese which emphasizes the need to address general parenting behavior in global primary prevention approaches.

## Figures and Tables

**Table 1:** Sample characteristics.

	Total Sample (*n* = 1518)	Male Students (*n* = 794)	Female Students (*n* = 724)	BMI ≤P90 (*n* = 1373)	BMI >P90 (*n* = 145)
**Age**					
Mean (*SD*)	14.90 (0.68)	14.99 (0.69)	14.81 (0.66)	14.90 (0.68)	14.97 (0.67)
Range	13–19	13–19	13–18	13–19	14–17
Welfare recipients	198 (13.0)	91 (11.5)	107 (14.8)	169 (12.3) **	29 (20)
Immigrant background	655 (43.1)	433 (43.3)	311 (43.0)	575 (41.9) **	80 (55.2)
**BMI**					
Mean (*SD*)	20.62 (3.04)	21.08 (3.22)	20.12 (2.74)	-	-
Range	12.96–39.06	14.96–39.06	12.96–33.30	-	-
>P90 (%)	9.6	12.0	6.9 **	-	-
**Parental Physical Abuse in Childhood**					
No (%)	805 (53.0)	426 (53.7)	379 (52.3)	741 (54.0)	66 (45.5)
Moderate (%)	527 (34.7)	270 (34.0)	257 (35.5)	472 (34.4)	52 (35.9)
Severe (%)	186 (12.3)	98 (12.3)	88 (12.2)	160 (11.7) *	27 (18.6)
**Parental Warmth in Childhood**					
Low (%)	130 (8.6)	63 (8.4)	63 (8.7)	115 (8.4)	13 (9.0)
Medium (%)	805 (53.0)	456 (57.4)	349 (48.2)	715 (52.1)	93 (64.1)
High (%)	583 (38.4)	271 (34.1)	312 (43.1) ***	543 (39.5)	39 (26.9)
**Parental Monitoring in Childhood**					
Low (%)	143 (9.4)	88 (11.1)	55 (7.6)	12 (8.8)	19 (13.1)
Medium (%)	866 (57.0)	476 (59.9)	390 (53.9)	776 (56.5)	92 (63.4)
High (%)	509 (33.5)	230 (29.0)	279 (38.5) ***	476 (34.7)	34 (23.4)

BMI, body mass index; BMI >P90, overweight/obese. Gender differences tested by means of chi-square. * *p* < 0.05; ** *p* < 0.01; *** *p* < 0.001.

**Table 2:** Zero-order correlations (Spearman rank order correlations).

	BMI >P90	PPA	PW	PM
BMI >P90		0.04	−0.09 **	−0.11 **
PPA	0.07		−0.32 ***	−0.18 ***
PW	−0.04	−0.36 ***		0.48 ***
PM	−0.01	−0.24 ***	0.45 ***	

Intercorrelations for males (*n* = 794) are presented above the diagonal, and intercorrelations for females (*n* = 724) are below the diagonal. BMI >P90, overweight/obese; PPA, Parental physical abuse in childhood; PW, Parental warmth in childhood; PM, Parental monitoring in childhood. For PV, PW, and PM, higher scores are indicative of more extreme responding. ** *p* < 0.01; *** *p* < 0.001.
